# Health-related SDGs in the national science agendas of Latin America and the Caribbean: a scoping review

**DOI:** 10.1186/s12939-024-02350-w

**Published:** 2025-06-16

**Authors:** Martín Alberto Ragusa, Fernando Tortosa, Maristela Monteiro, Sebastian Garcia Saiso, Ludovic Reveiz

**Affiliations:** Unit on Science and Knowledge for Impact of the Evidence and Intelligence for Action in Health Department, Pan American Health Organization (PAHO) , 525 23rd Street NW, Washington DC, 20037 USA

**Keywords:** National science technology and innovation policy, Innovation and development policy, Science technology and innovation management in health, Sustainable development, Disaster emergencies, Emergency surveillance

## Abstract

**Background:**

The national science and technology agendas (NSTAs) of Latin America and the Caribbean (LAC) are crucial for formulating and implementing public policies by providing a strategic framework that guides state actions and priorities. The objective of this scoping review is to examine health-related targets from the national science and technology agendas (NSTA) of Latin America and the Caribbean (LAC), in accordance with the United Nations’ third Sustainable Development Goal (SDG-3), as well as within the frameworks of innovation and risk management and emergencies.

**Methods:**

A scoping review was conducted, including policy documents issued between 2013 and 2023 by governmental science and technology authorities. The search strategy included government and international organization websites. A total of 108 documents were identified.

**Results:**

Sixteen NSTAs were selected. Health-related targets aligned with SDG-3 were highlighted, particularly in areas such as communicable diseases and drug and vaccine development, but there was limited representation in public health and health systems. Innovations in health science and technology included diagnostic technologies, health products and artificial intelligence. Risk management for health emergencies and disasters was present in a minority of the agendas, with a focus on natural disasters and the COVID-19 pandemic.

**Conclusions:**

This analysis provides a comprehensive view of the representation of health in NSTAs in LACs, highlighting common objectives among countries to foster collaboration, optimize research and innovation, and identify gaps in components necessary to enhance population health, such as disaster management, public health, and health systems.

**Registration:**

This scoping review was not registered.

## Background

National agendas are key tools for formulating and implementing public policies in Latin America and the Caribbean (LAC). They represent a strategic framework that guides the actions and priorities of states in various development areas, focusing on priority areas and facilitating coordination among different actors such as governments, civil society, the private sector, and international organizations to promote more effective and consensual policies [1]. Advances in science and technology generate the expectation of finding new and improved ways to address current and future health challenges and ensure healthier populations worldwide. The COVID-19 pandemic highlighted the importance of linking national science and technology agendas (NSTA) with health to create more resilient health systems and societies capable of facing current and future health challenges. The need to strengthen the relationship between science, technology, and health arises from the required focus on closing knowledge gaps to address national/local priorities and thus advance public health [2]. Science and technology are advancing rapidly, and the PAHO and the World Health Organization (WHO) are striving to understand and promote the latest advances in relevant areas of scientific research and technology to identify, anticipate, and prepare for problems affecting global health. The WHO’s Global Health Foresight function was created to help Member States incorporate “future thinking” into strategic health planning. Foresight monitors scientific and technological advances to support Member States in ensuring better risk anticipation, improved preparedness, and timely management by adopting and scaling scientific advances with potential for global health [3].

NSTAs in LAC countries can play a crucial role in developing and overcoming global health challenges and sustainable development by focusing on research, development, and innovation tailored to the specific needs of the population, which is essential for addressing unique health problems in these countries. Setting priorities in these agendas is essential to direct limited financial and human resources aimed at maximizing public health impact and strengthening national capacities, which in turn drives local scientific and technological progress. By respecting the national political space to establish distinctive policies for sustainable development, NSTAs also contribute to achieving the SDGs [4].

To provide a comprehensive idea of how health is represented in the national scientific agendas of LAC countries, this scoping review aims to identify and present available information on health targets on national policy agendas, strategies, and science plans of Latin American countries following three predefined conceptual frameworks: (1) The targets of SDG-3 [4]; (2) The global horizon scan of advances to improve global public health was developed by the WHO [3]; and (3) The WHO Health Emergency and Disaster Risk Management Framework [5].

A scoping review was chosen as the study methodology because mapping health objectives represented in scientific policy agendas can be used to define research priorities and frameworks, clarify concepts, and inform future synergies between the Ministries of Science, Technology, Innovation, and Health of Member States and the PAHO/WHO.

## Methods

This scoping review was written according to the methodology of the Joanna Briggs Institute [6].

### Eligibility criteria

Agendas were selected on the basis of the following attributes: (i) formulated by governments on behalf of the “public”; (ii) structured as a response to a problem and oriented toward a desired state or goal to solve the problem; and (iii) implemented and interpreted by public and private actors who have diverse interpretations of solutions and problems [7].

### Inclusion criteria

- Issued between January 1, 2013, and May 31, 2023, or if issued before 2013, did not expire before 2018 (last 5 years).
- Published by national government authorities responsible for science and technology in LAC countries at the national level.

### Exclusion criteria

- The full texts of the documents were not publicly available.
- Subnational scope documents.

### Information sources and search strategy

The search was conducted on government websites; the Regional Observatory of Planning for Development in Latin America and the Caribbean of the Economic Commission for Latin America and the Caribbean (ECLAC); the United Nations Educational, Scientific and Cultural Organization (UNESCO) Information System on Educational Trends in Latin America (SITEAL); the United Nations Development Programme (UNDP); the United Nations Conference on Trade and Development (UNCTAD); the World Bank; the Organization for Economic Cooperation and Development (OECD); the United Nations Environment Programme (UNEP); and Google. The keywords used to identify policy documents were “science”, “ciencia” and “ciência”.

### Selection process and management of identified documents

Potentially relevant documents were first screened at the title and/or abstract level by one author, followed by a full-text examination of sources that appeared relevant independently by two authors for selection. Discrepancies were resolved by an external reviewer. Finally, efforts were made to contact governments to share the found documents and request additional documents if not found during the search phase. An adapted PRISMA-ScR flowchart was used to graphically represent the movement of sources through the search process to their eventual inclusion [6].

### Data extraction

Essential information, including country, document name, source, publication date, time horizon, and health-related objectives/goals, was extracted into Microsoft Excel. For the latter, the text of each health objective from the original policy document was transferred to the extraction form.

### Analytical framework

The health goals from the documents were “mapped” via three frameworks:

1. SDG-3 targets [4]. In 2015, the United Nations approved the 2030 Agenda for Sustainable Development. The Agenda has 17 Sustainable Development Goals that state that poverty eradication must go hand in hand with strategies that foster economic growth and address a range of social needs, including education, health, social protection, and job opportunities, while addressing climate change and environmental protection. The third goal aims to ensure healthy lives and promote well-being for all ages and is divided into 13 targets.
2. WHO’s global horizon scan for health innovations [3]. In 2022, the WHO’s Science Division initiated a horizon scanning exercise to identify innovations in science and technology that could improve global health, including addressing health needs that are frequently neglected or inadequately addressed. The exercise is part of the discipline of “foresight,” which involves exploring, anticipating, and shaping the future to build and use collective intelligence in a structured and systematic way to anticipate developments. The objective was to identify innovations in areas of research and emerging technologies and their potential uses and opportunities to gain useful insights for strategic planning, policy formulation, and preparedness. It is divided into seven groups and multiple subgroups.
3. WHO’s Health Emergency and Disaster Risk Management Framework [5]. The health emergency and disaster risk management framework provides a common language and a comprehensive approach that can be adapted and applied by all health sector and other sector actors working to reduce health risks and the consequences of emergencies and disasters. The framework aims to improve health outcomes and the well-being of at-risk communities in different contexts, particularly in fragile settings with both low and high resources. Specifically, we used the component of information and knowledge management, which consists of five functions: risk assessment; early warning and surveillance; research on health emergencies and disaster risk management; knowledge management (guidance and technical support); and information management.

### Analysis and presentation of data

Basic descriptive frequency analyses were conducted to present the results of the essential extracted information and the analytical framework through the iterative development and selection of figures and tables.

## Results

### Identification of agendas

A total of 108 documents were identified, 30 of which were selected for full-text review. Science- and technology-specific documents were identified for the following countries: Argentina [8], Brazil [9], Bolivia [10], Chile [11], Colombia [12], Costa Rica [13], Cuba [14], Ecuador [15], El Salvador [16], Guatemala [17], Honduras [18], Jamaica [19], Mexico [20], Nicaragua [21], Panama [22], Paraguay [23], Peru [24], the Dominican Republic [25], Uruguay [26], and Venezuela [27]. National development plans with some sections dedicated exclusively to science and technology were identified for Antigua and Barbuda [28], Belize [29], and Dominica [30]. No national documents addressing a science and technology plan were identified for the remaining LAC countries.

Thirteen documents were excluded because of a lack of reference to health objectives or goals and/or inability to map with the predefined frameworks and one because it was outdated before 2018.

**Fig. 1:**
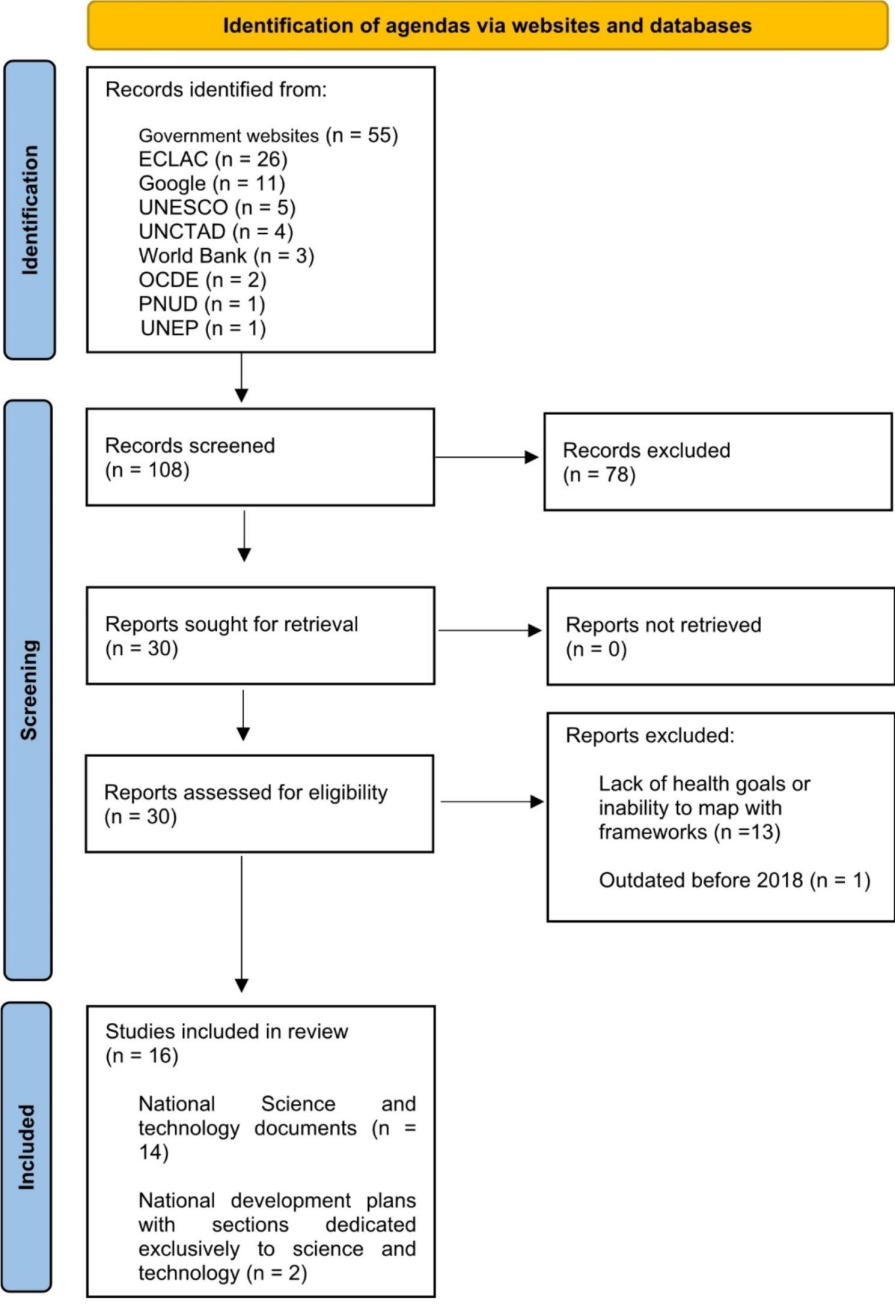
PRISMA-ScR flowchart

Sixteen countries’ agendas presented at least one health-related objective or goal that met the eligibility criteria: Antigua and Barbuda, Argentina; Belize, Brazil; Bolivia, Chile; Colombia; Costa Rica; Cuba, Ecuador; El Salvador; Panama; Paraguay; Peru; and Venezuela. As of the writing of this manuscript, the agendas of Argentina, Bolivia, Colombia, Costa Rica, Cuba, Ecuador, El Salvador, Panama, Paraguay, and Venezuela remain current. (Fig. 1)

### Analysis of agendas

#### Targets related to SDG-3

The agendas of 13 countries presented at least one objective or goal related to SDG-3 targets: Argentina, Brazil, Belize, Bolivia, Chile, Colombia, Costa Rica, Ecuador, El Salvador, Panama, Peru, and Venezuela (Table 1). Most agendas mentioned support for the development of drugs and vaccines and addressing communicable and noncommunicable diseases, whereas goals focusing on public health and health systems such as universal coverage and prevention of traffic injuries were underrepresented.

**Table 1:** Goals and objectives related to SDG-3

SDG-3 Target	Description	No. of Agendas	Countries (Target Year)
3.1	Reduce global maternal mortality	3	Ecuador (2030), Panama (2024), Peru (2021)
3.2	End preventable deaths of newborns and children under 5 years of age	4	Ecuador (2030), El Salvador (2030), Panama (2024), Peru (2021)
3.3	End the epidemics of AIDS, tuberculosis, malaria, and neglected tropical diseases and combat hepatitis, water-borne diseases, and other communicable diseases	9	Argentina (2030), Bolivia (2025), Brazil (2022), Chile (2022), Ecuador (2030), El Salvador (2030), Panama (2024), Peru (2021), Venezuela (2030)
3.4	Reduce premature mortality from non-communicable diseases	7	Argentina (2030), Bolivia (2025), Brazil (2022), Ecuador (2030), Panama (2024), Peru (2021), Venezuela (2030)
3.5	Strengthen the prevention and treatment of substance abuse	1	Panama (2024)
3.6	Halve global deaths and injuries from road traffic accidents	1	Panama (2024)
3.7	Universal access to sexual and reproductive health-care services	2	Ecuador (2030), Venezuela (2030)
3.8	Universal health coverage	2	Argentina (2030), Ecuador (2030)
3.9	Reduce deaths and illnesses from hazardous chemicals and air, water, and soil pollution and contamination	3	Belize (2019), Panama (2024), Peru (2021)
3.a	Strengthen the implementation of the World Health Organization Framework Convention on Tobacco Control	1	Panama (2024)
3.b	Research and development of vaccines and medicines	11	Argentina (2030), Bolivia (2025), Brazil (2022), Colombia (2026), Costa Rica (2027), Cuba (2025), Ecuador (2030), Peru (2021), Venezuela (2030)
3.c	Health financing and the recruitment, development, training, and retention of the health workforce	1	Ecuador (2030)
3.d	Strengthen the capacity for management of national and global health risks	4	Belize (2019), Chile (2030), Ecuador (2030), Panama (2024)

#### Targets related to innovations in health science and technology

The agendas of 12 countries presented at least one objective or goal related to innovations in health science and technology: Argentina, Brazil, Ecuador, El Salvador, Chile, Colombia, Costa Rica, Cuba, Panama, Paraguay, Peru, and Venezuela (Table 2). Most often, the objectives were more general than those presented in the WHO’s identified subgroups or were directly proposed without specific mention of human health.

**Table 2:** Goals and objectives related to innovations in health science and technology

Group	Areas of Interest	No of Agendas	Countries	Highlighted WHO Groups and Subgroups
1	Diagnostic Technologies	7	Argentina, Brazil, Costa Rica, Cuba, Panama, Paraguay, Peru	Diagnostic methods, communicable diseases, sensors, remote diagnosis, genomics, metagenomics
2	Health Products and Drug Delivery Technologies	9	Argentina, Brazil, Colombia, Costa Rica, Cuba, Ecuador, Panama, Peru, Venezuela	Bioeconomy, drug development, biologics/biotechnologies, 3D printing
3	Tissue Engineering and Regenerative Medicine	3	Brazil, Colombia, Peru	Advanced therapies, biochips
4	Molecular Biology, Cell, Immune, and Gene Therapy	3	Brazil, Colombia, Peru	Cell therapy, stem cell-based therapy, advanced therapies, gene therapy
5	Public Health: Environment, Climate Change, Epidemiology and Surveillance, Nutrition and Health	8	Argentina, Brazil, Chile, Costa Rica, Ecuador, El Salvador, Panama, Peru	Environment, biosensors, telehealth, genomics, big data, drones, territorial mapping
6	Dissemination and Implementation	6	Argentina, Brazil, Colombia, Costa Rica, Ecuador, Peru	Digital technologies, health care systems and services
7	Artificial Intelligence, Internet of Things, Wearables, Telehealth, Augmented and Virtual Reality	5	Argentina, Chile, Costa Rica, Brazil, Ecuador	AI, Internet of Things, quantum encryption, cybersecurity
8	Prosthetic Materials and Biomaterials	3	Argentina, Brazil, Costa Rica	Materials research, sensors, 3D printing

#### Targets related to health emergency and disaster risk management

The agendas of nine countries presented at least one objective or goal related to health emergency and disaster risk management: Antigua and Barbuda, Argentina, Belize, Brazil, Chile, Ecuador, El Salvador, Panama, and Peru (Table 3). No agendas with objectives applying to the knowledge management function were identified, and the elements for other functions were specifically covered for the environmental domain (natural disasters) or the COVID-19 pandemic.

**Table 3:** Goals and objectives related to health emergency and disaster risk management

Function	Country	Details
Risk Assessments	Panama	Risk assessment (no additional specifications).
	Argentina	Assessment of the impact of environmental and pollution variables on infectious diseases.
	Chile	Climate Change Observatory for natural laboratory data for Cloud Computing, artificial intelligence, and Data Science.
	Peru	Prospective studies for natural disaster prevention.
Early Warning and Surveillance	Antigua and Barbuda, and Belize	Disaster risk management and climate change resilience.
	Brazil	Consolidation of the security system for sector activities and emergency response.
	Ecuador	Development of methods, techniques, and approaches for risk prevention and mitigation.
	El Salvador	Early Warning Systems from the Ministry of Environment and Natural Resources.
	Peru	Prospective studies, prevention, and disaster mitigation.
	Chile	Data for predictive pandemic analysis (COVID-19 Data Subcommittee).
	Peru	Prospective studies, prevention, and disaster mitigation.
Knowledge Management	-	No agendas with related objectives identified.
Information Management	Chile	Data for predictive pandemic analysis (COVID-19 Data Subcommittee).
	Ecuador	Development of methods, techniques, and approaches for risk prevention and mitigation.

## Conclusions

The analysis of 16 NSTAs from LAC countries provides a general overview of how health goals, innovation, and risk management are represented in science and technology policy LACs. The findings presented in the summary highlight the intersection between science, technology, and health policy in the region. While alignment with SDG-3 is evident, there are notable gaps and opportunities for improvement requiring contextualized approaches and innovative solutions.

Most agendas referencing any of the SDG-3 targets highlight priorities such as stopping disease transmission, reducing noncommunicable diseases, and supporting research and development of vaccines and drugs, whereas other targets related to public health policies and health systems such as traffic injuries, sexual and reproductive health services, mental health, universal health coverage, environmental health, anti-tobacco measures, health financing, and training are scarcely mentioned.

The scarce representation of these targets may be due to multiple factors. A weak interconnection between science and technology officials and other sectors, such as health, economy, and the environment; the need to establish long-term and difficult-to-evaluate strategies; the intrinsic complexity of these policies; lack of public awareness; and potential inequality in resource distribution compared with more industry-oriented agendas, are potential causes of their limited representation in the selected agendas. To address these limitations and improve the representation of public health and health systems in NSTAs, it is crucial to raise public awareness about the importance of these areas, promote interdisciplinary collaboration, and advocate for long-term evidence-based policies [31].

We identified objectives related to each WHO scientific interest group. However, most often, the objectives were more general than those presented in the WHO’s identified subgroups or were directly proposed without specific mention of human health. This generic representation may be due to the broad scope of health innovation, encompassing areas from drugs to care models; the rapid pace of advancements; and the need for flexibility in the context of financial uncertainty by establishing general goals applicable to a variety of innovations. A more robust linkage to health investment agendas with the productive/industrial sector could strengthen the presence of WHO scientific interest group innovations in NSTAs specifically applied to human health.

Emergency or disaster management is part of some objective in only nine of the 16 identified agendas in the region. Even for the specific topics of risk management research, knowledge management, and information management, we found only one or two agendas mentioning that particular domain. The preventive, long-term, and less tangible nature of disaster management, by nature sporadic and difficult to predict, and the interdisciplinary conditions encompassing meteorology, civil engineering, and behavioral psychology are potential mechanisms for this limited representation of emergencies in science agendas.

The concordance found between several NSTAs is a key element in strengthening collaboration among countries in the Americas. To achieve this, it is necessary to identify and implement effective strategies to optimize investment in research, development, and innovation. Collaboration initially facilitates the exchange of knowledge and experiences among scientists and experts from different countries, thus enriching the quality of research and technological development and fostering innovation. Additionally, participation in collaborative projects could facilitate access to funds from international, regional, and national organizations to increase funding for research and innovative projects in the region’s countries, including the development of regional production platforms. Joint funding mechanisms can be used to improve scientific and technological infrastructures in countries, including advanced laboratories and state-of-the-art research centers [32]. PAHO highlights the vital importance of upholding high ethical standards in scientific research to enhance national ethics oversight systems and foster collaboration among health authorities, scientific agencies, and research institutions. It provides a robust framework designed to promote integrity in research partnerships and offers valuable resources on ethical collaboration. These efforts are aimed at reducing scientific misconduct and advancing reliable, high-quality health research across the region [33–35].

This review has the strength of being a novel document that can help identify gaps in relevant domains in science policies that seek to improve the health of populations in the Americas following robust and updated frameworks of specific goals, prioritized groups of innovations, and disaster prevention and response.

As limitations, we selected documents addressing NSTAs and did not conduct specific searches for each framework or component, which may have hindered the identification of relevant documents providing more details on a particular policy or objective. Future analyses could aim to identify documents with these specific components related to innovation, health goals, or some aspect of risk management. Owing to its novel nature, we can assert that the frameworks selected for innovation and emergency analysis were not considered when the agendas were drafted, which explains the lack of optimal combination between the agenda objectives and the selected framework components. Another limitation is the lack of assessment of how the agendas align with financial and policy commitments or intersectoral collaboration and the absence of comparative policy analysis. Limited resources, the challenges of coordinating diverse sectoral goals, and persistent governance issues may contribute to rendering the agendas primarily aspirational in nature. Future research should prioritize evaluating implementation strategies and assessing the tangible impacts of these agendas to strengthen the analysis of national and global commitments toward achieving SDG transformation.

The analyzed NSTAs can serve as inputs to identify and enhance synergies between Member States, PAHO/WHO, and other multilateral organizations to improve the health of the continent’s population.

In conclusion, while the analysis provides valuable information on the representation of health in science and technology agendas in LACs, there is room to strengthen the relevance of the health sector. By addressing the identified gaps and fostering collaboration, countries can better leverage science and technology to promote population health and advance the achievement of sustainable development goals.

## Data Availability

The data that support the findings of this study are not openly available but they are available from the corresponding author upon reasonable request.

## Abbreviations

ECLAC: Economic Commission for Latin America and the Caribbean
LAC: Latin America and the Caribbean
NSTA: National science and technology agendas
OECD: Organization for Economic Cooperation and Development
PAHO: Pan American Health Organization
SDG-3: United Nations’s third Sustainable Development Goal
SITEAL: System on Educational Trends in Latin America
UNCTAD: The United Nations Conference on Trade and Development
UNDP: The United Nations Development Programme
UNEP: United Nations Environment Programme
UNESCO: United Nations Educational, Scientific and Cultural Organization
WHO: World Health Organization

## References

[1] OECD, Technology and Innovation Outlook. (2023), OECD Science, 2023: Enabling Transitions in Times of Disruption, OECD Publishing, Paris, 10.1787/0b55736e-en

[2] A systematic approach for. Undertaking a research priority-setting exercise. Guidance for WHO staff. Geneva: World Health Organization; 2020.

[3] 2023 Emerging technologies and scientific innovations: a global public health perspective. Geneva: World Health Organization; 2023 (WHO global health foresight series).

[4] United Nations. The 2030 agenda and the sustainable development goals: an opportunity for Latin America and the Caribbean (LC/G.2681-P/Rev.3), Santiago, 2018.

[5] Emergency H. and Disaster Risk Management Framework. Geneva: World Health Organization; 2019.

[6] Tricco AC, Lillie E, Zarin W, O’Brien KK, Colquhoun H, Levac D, et al. PRISMA extension for scoping reviews (PRISMA-ScR): checklist and explanation. Ann Intern Med. 2018;169(7):467–73. 30178033 10.7326/M18-0850

[7] Klepac Pogrmilovic B, O’Sullivan G, Milton K, et al. A global systematic scoping review of studies analyzing indicators development and content of national-level physical activity and sedentary behavior policies. Int J Behav Nutr Phys Act. 2018;15(1):123. 10.1186/s12966-018-0742-9 30486826 10.1186/s12966-018-0742-9 PMC6263060

[8] Argentina. Plan Nacional de Ciencia, Tecnología e Innovación 2030. https://www.argentina.gob.ar/ciencia/seppcti/plan-nacional-de-ciencia-tecnologia-e-innovacion-2030

[9] Brasil. ESTRATÉGIA NACIONAL DE, CIÊNCIA, TECNOLOGIA E. INOVAÇÃO 2016–2022. https://bibliotecadigital.economia.gov.br/bitstream/123456789/990/1/ENCTI-MCTIC-2016-2022.pdf

[10] Bolivia. Plan Nacional de Ciencia, Tecnología e Innovación 2013–2025. https://siteal.iiep.unesco.org/sites/default/files/sit_accion_files/10265.pdf

[11] Chile. Política Nacional de Ciencia, Tecnología, Conocimiento e Innovación. PLAN DE ACCIÓN 2020–2022. https://www.minciencia.gob.cl/el-ministerio/politica-nacional-de-ctci/

[12] Colombia. Plan de Acción Institucional (PAI) 2023–2026. https://minciencias.gov.co/quienes_somos/planeacion_y_gestion/planeacion_gestion_pai_list

[13] Costa Rica, PLAN NACIONAL DE CIENCIA, TECNOLOGÍA E. INNOVACIÓN 2022–2027. https://www.micitt.go.cr/wp-content/uploads/2022/06/Plan_Nacional_Ciencia_Tecnologia_Innovacion_2022-2027.pdf

[14] Cuba. Programas Nacionales de Ciencia, Tecnología e Innovación 2021–2025. https://siteal.iiep.unesco.org/sites/default/files/sit_accion_files/cuba_decreto_ley_ndeg_7_del_2020_del_sistema_de_ciencia_tecnologia_e_innovacion_y_su_decreto_ley_reglamentario_ndeg_40_del_2021.pdf

[15] Ecuador. Plan Nacional de Ciencia, Tecnología, Innovación y Saberes Ancestrales 2021–2030. https://www.bivica.org/files/5879_Plan Nacional SENESCYT.pdf

[16] El Salvador. Política Nacional de Innovación, Ciencia y Tecnología 2018–2030. https://siteal.iiep.unesco.org/bdnp/3910/politica-nacional-innovacion-ciencia-tecnologia-2018

[17] Guatemala. Política Nacional de Desarrollo Científico y Tecnológico 2015–2032. https://www.senacyt.gob.gt/portal/attachments/legislacion/PoliticaNacionaldeDesarrolloCyT21062017.pdf

[18] Honduras, PLAN ESTRATÉGICO INSTITUCIONAL. IHCIETI 2016–2019. https://senacit.gob.hn/documentos/PLAN-ESTRATEGICO-INSTITUCIONAL-IHCIETI-2018.pdf

[19] Jamaica, National Science, Technology & Innovation Policy. 2022. SECTOR PLAN 2021–2030. https://www.mset.gov.jm/wp-content/uploads/2022/09/National-ST-I-Policy-2022-v2.pdf

[20] México. PROGRAMAINSTITUCIONAL, 2020–2024 DEL, CONSEJO NACIONAL DE CIENCIA Y. TECNOLOGÍA 2020–2024. https://conahcyt.mx/conahcyt/programa-institucional-2020-2024/

[21] Nicaragua. Política nacional de ciencia, tecnología e innovación 2011 – 2013. http://conicyt.gob.ni/wp-content/uploads/2016/01/politica-nacional-de-cienciay-tec-version-final.pdf

[22] Panamá. Plan Estratégico Nacional de Ciencia, Tecnología e Innovación (PENCYT) 2019–2024. https://www.senacyt.gob.pa/wp-content/uploads/2021/06/PENCYT_2019-2024_ACTUALIZACION-Y-PRIORIZACION-SECTORIAL.pdf

[23] Paraguay. Agenda Nacional de Ciencia, Tecnología e Innovación 2022–2030. https://www.conacyt.gov.py/sites/default/files/upload_editores/u489/Agenda-Nacional-CTI.pdf

[24] Perú. Plan Nacional Estratégico de Ciencia, Tecnología e Innovación para la competitividad y el desarrollo humano 2006–2021. https://www.gob.pe/institucion/concytec/informes-publicaciones/1326952-plan-nacional-estrategico-de-ciencia-tecnologia-e-innovacion-para-la-competitividad-y-el-desarrollo-humano-2006-2021

[25] República Dominicana. Plan Estratégico de Ciencia, Tecnología e Innovación 2008–2018. https://mescyt.gob.do/wp-content/uploads/2022/02/Plan_Estrategico_CyT.pdf

[26] Uruguay, PLAN ESTRATÉGICO NACIONAL DE CIENCIA, TECNOLOGÍA E. INNOVACIÓN 2010–2030. https://anii.org.uy/upcms/files/listado-documentos/documentos/pencti.pdf

[27] Venezuela. Plan Nacional de Ciencia, Tecnología e Innovación. Construyendo un futuro sustentable 2005–2030. https://siteal.iiep.unesco.org/sites/default/files/sit_accion_files/plan_nacional_de_ciencia_tecnologia_e_innovacion_2005-2030.pdf

[28] Antigua y Barbuda. Medium-Term Development Strategy 2016 to 2020. https://observatorioplanificacion.cepal.org/sites/default/files/plan/files/antigua_barbuda_medium_term_development_strategy.pdf

[29] Belize. Growth and Sustainable Development Strategy for Belize 2016–2019. https://observatorioplanificacion.cepal.org/sites/default/files/plan/files/BelizeGSDS.pdf

[30] Dominica. National Resilience Development Strategy Dominica. 2030. https://observatorioplanificacion.cepal.org/sites/default/files/plan/files/Dominica 2030The National Resilience Development Strategy.pdf

[31] Organización Panamericana de la Salud. (2022). Guía para la toma de decisiones informada por la evidencia incluso en las emergencias de salud OPS/EIH/KT/COVID-19/21–038

[32] Economic Commission for Latin America. and the Caribbean (ECLAC), science, technology and innovation: cooperation, integration and regional challenges (LC/TS.2022/156), Santiago, 2023.

[33] Pan American Health Organization. Catalyzing ethical research in emergencies. Ethics Guidance, Lessons Learned from the COVID-19 Pandemic, and Pending Agenda. Washington, D.C.: PAHO. 2022. https://iris.paho.org/handle/10665.2/56139

[34] Saenz C, Krahn TM, Smith MJ, Haby MM, Carracedo S, Reveiz L. Advancing collaborative research for health: why does collaboration matter? BMJ Glob Health. 2024;9(9):e014971. 10.1136/bmjgh-2024-014971 39284676 10.1136/bmjgh-2024-014971 PMC11409266

[35] Pan American Health Organization. PAHO supports over ten countries in Latin America and the Caribbean to improve ethical oversight of clinical trials. PAHO. 2024, November 5. https://www.paho.org/en/news/5-11-2024-paho-supports-over-ten-countries-latin-america-and-caribbean-improve-ethical

